# Partial and Total Flap Failure after Fibula Free Flap in Head and Neck Reconstructive Surgery: Retrospective Analysis of 180 Flaps over 19 Years

**DOI:** 10.3390/cancers13040865

**Published:** 2021-02-18

**Authors:** Michael Knitschke, Sophia Sonnabend, Christina Bäcker, Daniel Schmermund, Sebastian Böttger, Hans-Peter Howaldt, Sameh Attia

**Affiliations:** Department of Oral and Maxillofacial Surgery, Justus-Liebig-University, Klinikstrasse 33, 35392 Giessen, Germany; Sophia.M.Sonnabend@dentist.med.uni-giessen.de (S.S.); Christina.Baecker-2@dentist.med.uni-giessen.de (C.B.); Daniel.Schmermund@uniklinikum-giessen.de (D.S.); Sebastian.Boettger@uniklinikum-giessen.de (S.B.); HP.Howaldt@uniklinikum-giessen.de (H.-P.H.); Sameh.Attia@dentist.med.uni-giessen.de (S.A.)

**Keywords:** fibula free flap, head and neck cancer, reconstructive surgery, FFF success rate, FFF failure rate

## Abstract

**Simple Summary:**

Most data concerning fibula free flaps after cancer resection in the head and neck region are limited to small sample sizes and a short period of follow-up. This retrospective study aims to evaluate the flap success, failure, and complications at the recipient site in 180 cases over 19 years. The flap failure is classified as partial and total flap necrosis. A correlation between flap failure and patients’ medical status, age, sex, BMI, ASA-Score, planning type, and reconstruction time point was performed. Our findings help head and neck surgeons understand the factors that influence flap failure and assess risk factors. Our observations could optimize the treatment of cancer patients receiving a fibula free flap in the future.

**Abstract:**

Fibula free flap (FFF) is widely used in head and neck reconstructive surgery and is considered as a standard and therapy of choice after ablative cancer surgery. The aim of this retrospective monocenter study was to determine the success rates of fibula free flaps for jaw reconstruction after ablative tumor surgery. The disease course of patients who underwent jaw reconstructive surgery with FFF from January 2002 to June 2020 was evaluated regarding the flap success rate. Flap failure was analyzed in detail and categorized into two groups: partial flap failure (PFF) and total flap failure (TFF). A total of 180 free fibular flaps were performed over the last 19 years and a total of 36 flap failures were recorded. TFF occurred in *n* = 20 (56.6%) and PFF in *n* = 16 cases (44.4%) cases. No statistically significant differences were found concerning patients’ age at flap transfer, sex, BMI, ASA-Score, preoperative non-virtual or virtual surgical planning (non-VSP vs. VSP), and time of reconstruction (immediately vs. delayed). Duration of hospitalization shows statistically significant differences between both groups (*p* = 0.038), but no differences concerning operating time and duration on Intensive Care Unit (ICU). Partial flap failure appears to be underreported in literature. Sub- and complete failure of the skin paddle leads to clinical complaints like uncovered bone segments and plate exposure. Partial or complete FFF failure lead to infections on the recipient site and prolonged wound healing and therefore may cause a delay of the beginning of adjuvant radiation therapy (RT). PFF of hard tissue can be induced by RT.

## 1. Introduction

Since the first mandibular reconstruction with a fibula free flap (FFF) by Hidalgo in 1989, it has been shown that FFF is a reliable and versatile graft [1,2]. Currently, FFF is considered as standard therapy in head and neck reconstructive surgery, providing the optimal precondition for dental implant success and therefore for oral and dental rehabilitation [3,4]. Long-term complications on the donor-site are relatively low. Most patients have been satisfied with the functional and aesthetic results [5,6]. The number of free tissue transfers of soft and/or bone tissue defects have increased significantly in recent years [7]. The flap provides the opportunity to include a septo-cutaneous skin paddle of up to 200 cm^2^. Cadaver studies investigating skin perfusion through selective injection have shown that a skin area of 12 × 7 cm can be perfused by a single perforating vessel [8]. A recent milestone in operative techniques is the possibility of computer-assisted surgery (CAS) and virtual surgical planning (VSP) in reconstructions of jaws [9,10,11]. VSP initially focused on bone grafts. The innovative potential lies in the design of a cutting guide that takes into account the course of the cutaneous perforating vessels based on preoperative CT and sonographic measurements [12,13]. For skin paddle harvesting, the support of perforator vessels is crucial. Fibular flaps have been reported to allow a success rate of up to 95% [7,14,15,16]. The causes of flap failure are anastomosis insufficiency, more frequent venous congestion (e.g., edema, hematoma), rare arterial occlusion (e.g., embolism, thrombus, kinking), vasospasms, postoperative bleeding, and coagulopathies [17,18,19,20]. 

However, detailed data about partial FFF loss are rare and seem to be underreported in literature. An analysis of risk factors for flap failure and complications in a low-level center of 129 FFFs over a time span of 20 years reported a PFF rate of 7.8%. By definition, (sub-)total flap loss describes the failure of the skin paddle and/or the loss of one or more bone graft segments in poly-segmental reconstructions [21]. The data published to date lack a differentiation concerning soft and/or hard tissue loss. A comparative investigation on computer-assisted versus conventional FFF technique for craniofacial reconstruction found six skin paddle and two segmental graft failures, which corresponded to a PFF rate of 11.76% (*n* = 8 out of 68) and a TFF rate of 4.41% (*n* = 3) [22]. Comparative results of partial loss were reported with a range of 3–14% [16,23]. This should be considered when the often-described advantage of the skin paddle as a vital monitor is advocated [24,25]. Gennaro et al. mentioned that a thin muscle cuff around bone, e.g., the fibula or vascularized iliac crest bone flap, is needed for flap harvesting. In these cases, a direct clinical assessment even without the use of a Doppler probe is advisable [26]. However, if PFF or TFF occurs, therapeutic options and decisions must be made to reduce local infection, functional impairment, and increase the patient’s quality of life. In this study, we have assessed and discussed the therapeutic options in the setting of TFF.

The aims of this study are:

1. to estimate the rate of partial flap loss ((sub-)total loss of skin paddle and/or failure of graft segments) and total flap failure over a long time period of 19 years, and to suggest further therapy procedures according to localization and defect type;
2. to examine a correlation between age at flap transfer, BMI, ASA-Score, and risk factors in terms of partial and total flap failure; and
3. to investigate whether there is a correlation between non-VSP vs. VSP and time of reconstruction (immediate vs. delayed).

## 2. Materials and Methods

### 2.1. Study Design and Patient Population

**Figure 1:**
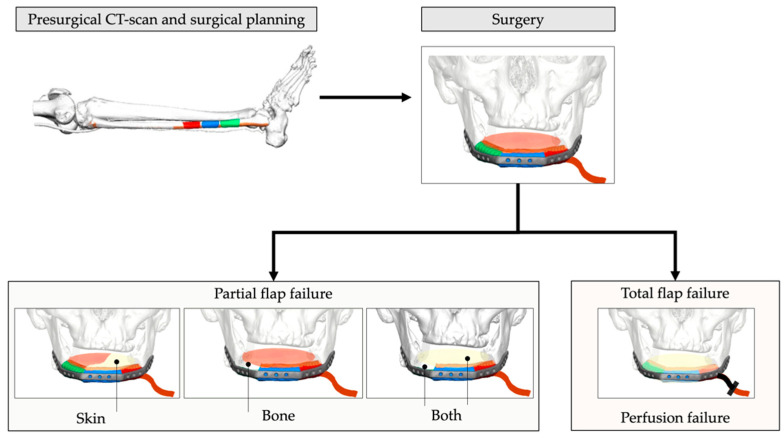
Schematic illustration of reconstructive workflow and stratification in partial and total flap failure groups. The major characteristic of PFF is the remaining blood supply by the vascular pedicle. In contrast to this, TFF is characterized by interrupted graft perfusion.

**Figure 2:**
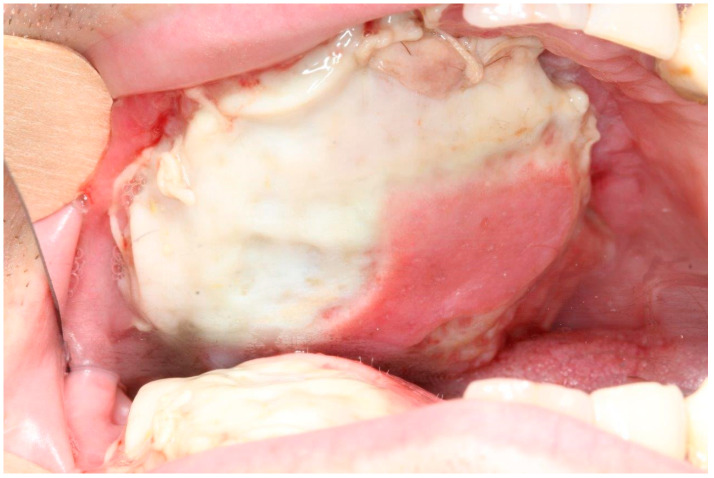
Example of ‘PFF skin’ after maxilla reconstruction.

**Figure 3:**
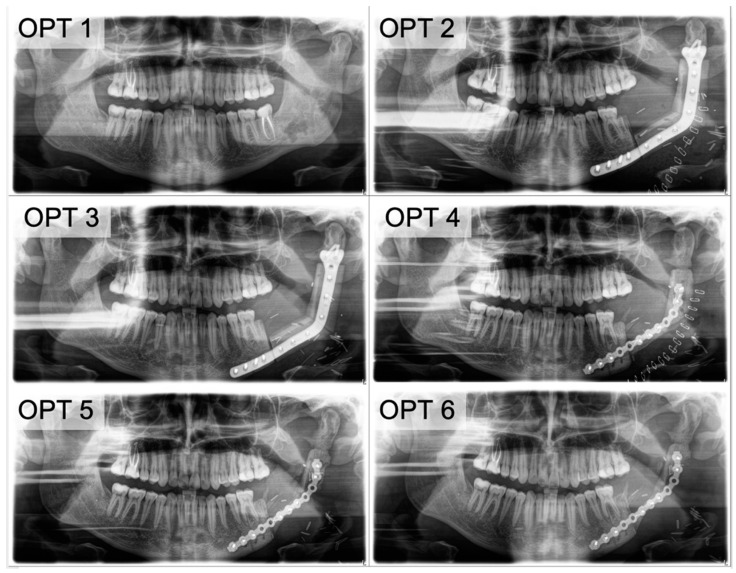
The clinical course of PFF. (**OPT 1**) Due to the recurrence of an ossifying fibroma (odontogenic tumor) in the ramus ascendens mandibulae in a 22-year-old patient, (**OPT 2**) a continuity resection and simultaneous reconstruction with a bi-segmental fibula and CAD/CAM plate was planned. (**OPT 3**) The clinical submandibular fistula had a connection to the plate (Figure 4A). (**OPT 4**) An OPT image was obtained after removal of the avital fibular segments and re-stabilization of the remaining graft. There is initial evidence of incipient bone healing on the proximal resection-site. (**OPT 5**) Follow-up visit at 9 weeks after re-stabilization. In the condylar segment, bone healing is pictured, and at the distal segment, progressive resorption is visible. (**OPT 6**) Progressive bone healing and callus formation originating from the resection site is visible 19 weeks after re-stabilization.

The study was conducted as a monocentric, retrospective study. Medical records of all patients who underwent FFF in the head and neck region from January 2002 to June 2020 were analyzed in respect of success of the flap transfer procedure. Flap failure was stratified into two groups: partial flap failure (PFF) and total flap failure (TFF) (Figure 1). PFF was defined as any loss of parts of the skin paddle (Skin) (Figure 2), parts or segments (poly-segmental reconstruction) of bone grafts (Bone) (Figure 3), or a combination of both (Both). The major characteristic of PFF is the remaining blood supply by the vascular pedicle. In contrast to PFF, TFF is characterized by discontinued perfusion of the graft (i.e., thrombosis). Intra- or extraoral wound dehiscences around the skin paddle alone did not match the criteria for PFF and were not included. 

All patients underwent a preoperative CT or MRI angiography to ensure the presence of a three fibular vessel anatomy and the absence of significant arteriosclerotic changes in the lower leg. Free fibula flap dissection was performed by Gilbert’s lateral approach [27]. To preserve knee and ankle stability, respectively, a bone length of 8 cm proximal to the harvest site and a distal bone length of 6–8 cm of distal to the harvest site were left in situ. When an osseo-cutaneous free flap was harvested, a muscle cuff with parts of soleus and flexor hallucis longus muscle was included to protect the perforators. Before donor-site wounds were closed, a vacuum drainage (Redon) was installed. Wound closure at the lower limb was performed using a split skin graft over the harvested skin flap or primarily in all cases of sole bone flaps without skin paddles.

**Figure 4:**
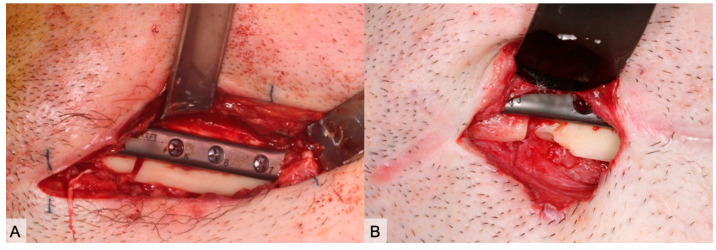
(**A**) Clinical aspect 4 weeks after reconstruction. After reopening the submandibular approach and excision of the skin fistula, there was no evidence of screw loosening. Temporary unscrewing of the screw (No. 5) leads to bleeding. (**B**) With a new fistula, the site was reentered 8 weeks after surgery and the screw was removed again. There was no clear bleeding from the screw hole. In addition, the anterior-caudal edge of the bone was removed.

**Figure 5:**
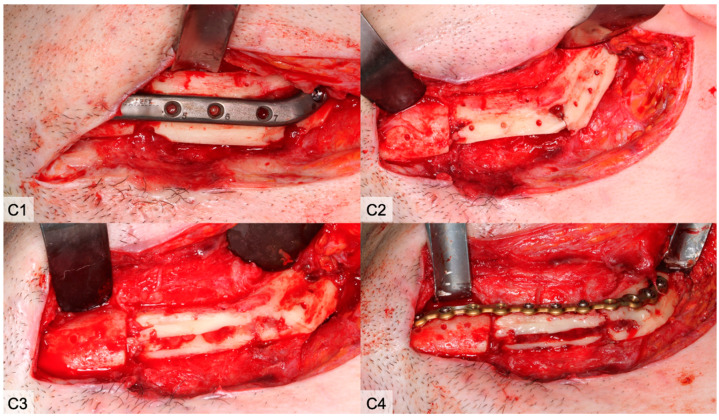
(**C1**) The CAD/CAM plate was removed 14 weeks after surgery when the fistula was productive again. (**C1**–**C3**) A sharp demarcation line caudal to the crestal edge of the plate became visible and was conspicuous. (**C2**) Between the fibula segments, an incipient ossification of the gap was observed. The underlying bone was pale. (**C3**) The bone was removed caudally. The bone marrow was replaced by granulation tissue and was removed. (**C4**) The intersegmental ossification was not yet sufficient so that stabilization again was necessary.

**Figure 6:**
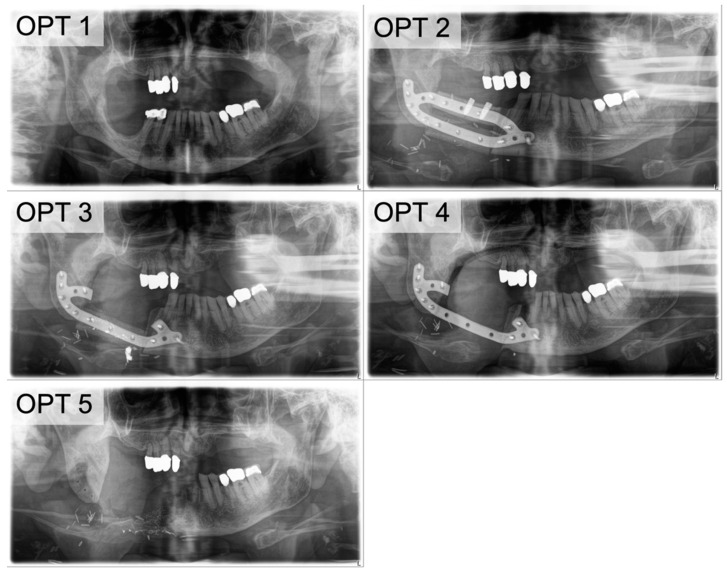
The following OPTs outline the clinical course of TFF in a 76-year-old patient in good general condition who suffered from osseous metastasis of prostate cancer and underwent FFF. (**OPT 1**) A pathologic fracture occurred after the removal of necrotic bone in the right lower jaw in the setting of bisphosphonate induced MRONJ stage III (treatment with Zoledronacid/Denosumab in prostate cancer) [28]. (**OPT 2**) Virtual planning of double-barrel fibula was performed. CAD/CAM plate was used for stabilization and the simultaneous insertion of two dental implants was performed. (**OPT 3**) At 3 weeks post-surgery, the skin paddle was lost. A surgical exploration ended with the removal of an avital distal graft segment and modification of the plate in situ. The vascular pedicle of the remaining FFF still rendered a clear signal in the Doppler ultrasonic probe. (**OPT 4**) At 16 weeks after surgery, the remaining graft segment also had to be removed in the setting of continued inflammation and the absence of a Doppler signal. (**OPT 5**) At 22 weeks after surgery: The screws loosened in the anterior mandible segment so that the remaining plate with teeth 32–42 and a bone sequester had to be taken out.

Examples for the clinical course of a subtotal bone graft loss (PFF) were illustrated in Figure 3, Figure 4 and Figure 5 and an example of TFF is demonstrated in Figure 6.

### 2.2. Study Parameters and Evaluator Calibration

The following parameters were collected: Patient age at the time of flap transfer, sex, primary diagnosis, planning procedure, location, type of defect classified according to Brown et al. [29,30], number of fibula segments, reconstruction time (immediately vs. delayed), flap condition, part of flap loss, and reason for flap loss. Patients’ medical records were analyzed independently for flap outcomes. 

### 2.3. Inclusion and Exclusion Criteria for Study Subjects

In this study, we enrolled all patients who underwent a reconstruction of the maxilla or mandible (immediately or delayed) with a FFF. Only cases with incomplete data sets and/or medical records were excluded (*n* = 2).

### 2.4. Statistical Analyses

Fischer test and Freeman–Halton extension [31] were used to compare flap outcome with sex, ASA-Score, alcohol and tobacco abuse, time and method of reconstruction, and the number of fibular bone segments. Students t-test was performed to compare the mean age at FFF-transfer, operating time, duration in the ICU, and time of hospitalization between the three flap outcome groups after verification of normality. *p* < 0.05 was defined as statistically significant. The statistical analysis was carried out with SPSS 25 (SPSS Inc., Chicago, IL, USA).

### 2.5. Ethics Statement/Confirmation of Patients’ Permission 

The study was approved by the local Ethics Committee of the Justus-Liebig University Giessen (AZ35/20) and patients’ consent was not necessary for this retrospective study. The patients consented that their intraoral pictures and X-ray images may be used anonymously in the publication. All data in the Microsoft Excel spreadsheet were pseudonymized.

## 3. Results

A total of 180 fibula free flaps (FFF) were performed over a period from January 2002 to June 2020. Complete flap success was recorded in 144 cases (80.0%). The remaining 36 flap failures were categorized into the two major groups partial (PFF) and total flap failure (TFF). PFF occurred in *n* = 16 (44.4%) and TFF in *n* = 20 (56.6%) cases (Table 1). No statistically significant difference concerning the age at flap transfer, sex, time, and method of reconstruction was apparent. Furthermore, no significant differences were detected in relation to surgical parameters (neck dissection, tracheostomy, radiation therapy) and general risk factors (alcohol and tobacco abuse). There is a significant difference concerning the duration of hospitalization between the groups PFF (mean 22.6 ± 9.7 days) and TFF (mean 33.8 ± 18.8 days) (*p* = 0.038). No statistically significant differences concerning operating time and duration in the ICU between PFF and TFF were detected. 

While TFF occurred in a median 8.5 days, PFF was clinically incident later. All TFF were an early complication. Out of 20 TFF (100%), four arterial and four venous thrombosis were found during anastomosis revision. The aetiology of the flap failure of the remaining 12 cases (60%) are unknown. PFF of the skin paddle (*n* = 11) was observed in a median time of 22.5 days and therefore considered as a late complication. The onset of PFF of bone segments without skin paddles (*n* = 4) was detected much later at a median time of 101.5 days. 

PFF was analyzed in detail. After maxillary reconstruction, two partial and three total flap failures were found in the investigation. This corresponds to 13.5% (*n* = 5) of the study collective. Two cases of PFF of the skin paddle was found after maxilla reconstructions (Brown Class II and III) with uni-segmental fibular, and three TFF were observed in uni- (*n* = 2) and bi-segmental reconstruction (*n* = 1). 

In mandible reconstruction, PFF was observed in 87.5% of cases. A total number of 14 partial flap failures—nine losses of the skin paddle, four isolated bone graft losses, and one combination of both—were found in mandible reconstructions after tumor recurrence (Table 2). Out of 11 skin paddle losses (PFF), 81.81% (*n* = 9) occurred in the conventional non-VSP group. 

**Figure 7:**
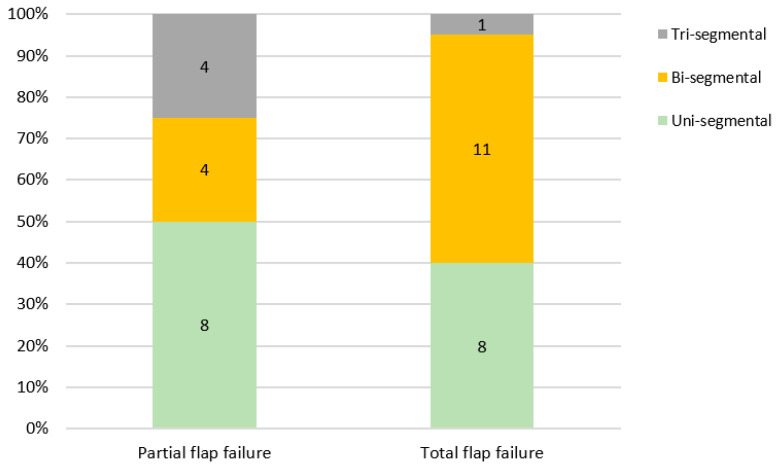
Partial or total flap failure in relation to the number of fibula segments (*p* = 0.114).

TFF was observed mostly in Brown class I (*n* = 7) and II (*n* = 6) defects. Subtotal loss of the skin paddle was incident in 68.7% of cases (*n* = 11 out of 16). Isolated loss of fibular bone segments was found in 25.0% (*n* = 4) and a combination of both was reported in only one case (6.3%). PFF was found in 50.0% (8 out of 16 cases) of uni-segmental, 25.0% (*n* = 4) of bi-segmental, and 25.0% (*n* = 4) of tri-segmental jaw reconstructions. A total flap loss occurred in 55.0% of cases after bi-segmental and in 40.0% of cases after uni-segmental reconstructions (Figure 7). There was a non-significant trend towards TFF in poly-segmental reconstructions (*p* = 0.114).

**Figure 8:**
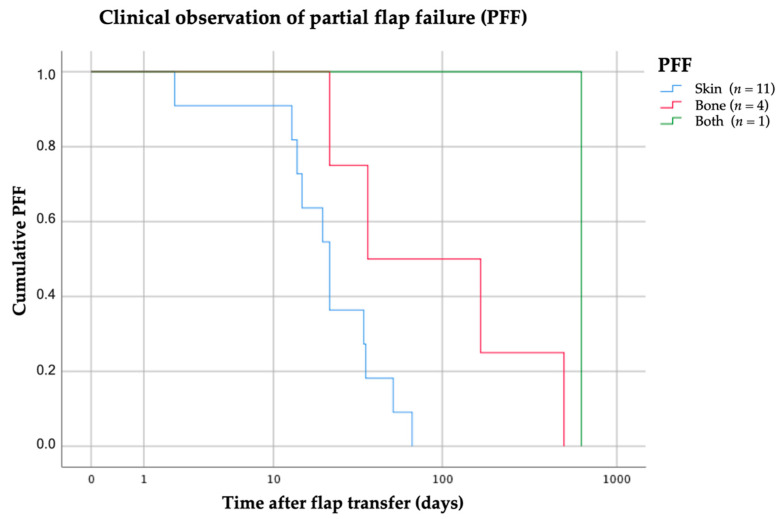
Kaplan–Meier function was drawn for the three sub-groups of PFF. The abscissa axis “time” (days) is drawn on a logarithmic scale.

A total loss of the skin paddle (*n* = 11) was observed at a mean of 27 days (median 22 days, range 2–67 days) and an isolated loss of bone graft segments at 181 days (*n* = 4; median: 101.5 days, range 37–499 days) post surgery. Kaplan–Meier survival function was calculated and visualizes the different periods of partial soft and hard tissue failure (Figure 8).

**Figure 9:**
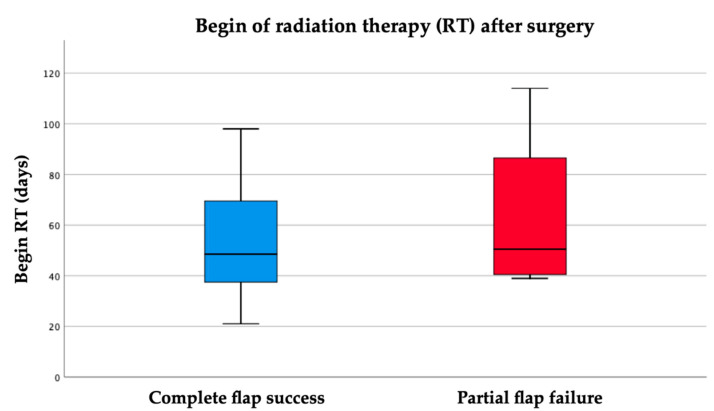
Comparison of the onset of adjuvant radiation therapy between the groups of complete flap success (*n* = 40) and PFF skin (*n* = 4).

In comparison to the beginning of radiation therapy in the complete success group (*n* = 40, mean 52.9 days, median 49 days, range 21–98 days), PFF of the skin paddle generates a delay (Figure 9). Radiation therapy started at a mean of 63.5 days (*n* = 4, median 50.5 days, range 39–114 days). The difference remains without any statistical significance (*p* = 0.358).

**Figure 10:**
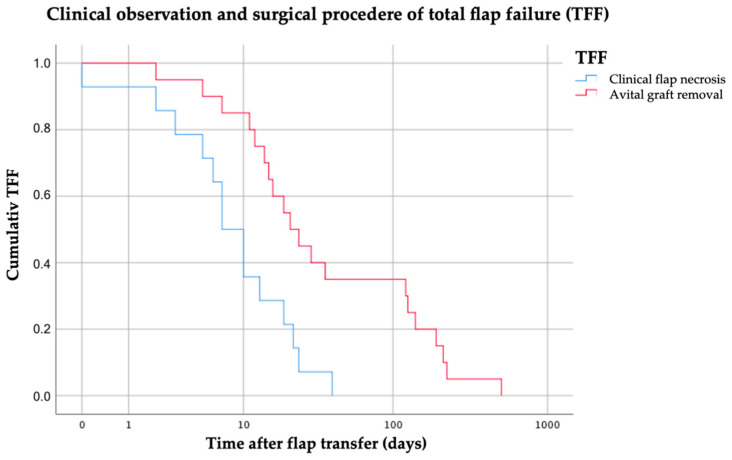
TFF concerning the first clinical signs of flap necrosis and time of removal of the avital graft was visualized by Kaplan–Meier function. The abscissa axis “time” (days) is drawn on a logarithmic scale.

Clinical flap necrosis and time of bone graft removal were compared with the number of bone segments used. Statistical analysis showed no significant differences between the uni- and bi-segmental graft losses. Overall, clinical flap loss was observed at a mean of 12 days after surgery (Median 8.5 days, range 0–40 days) and surgical removal of the avital grafts at a mean of 86.4 postoperative days (Median 22.5 days, range 2–503 days) (Figure 10).

**Figure 11:**
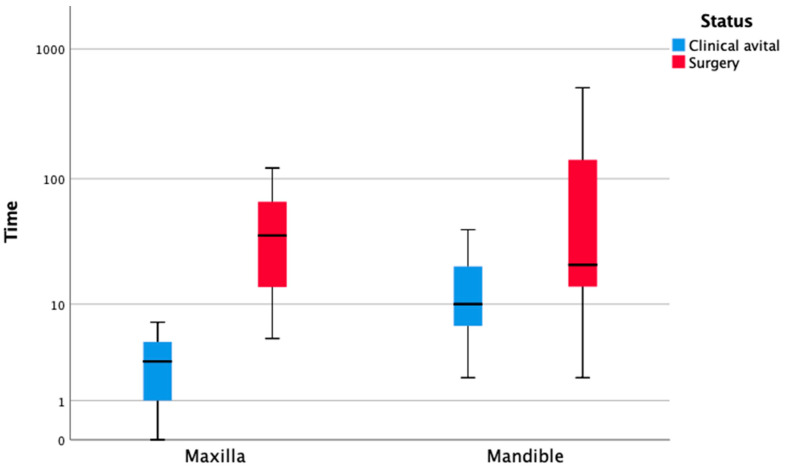
TFF analysis. Clinically diagnosed flap necrosis and further surgical interventions (removal of graft) are shown according to jaw location (maxilla or mandible) in the postoperative time course. The ordinate axis “time” (days) is drawn on a logarithmic scale.

Comparing TFF after maxillary and mandibular reconstruction, it is noticeable that maxillary TFF cases were a mean of 73.3 years old, and thus older than mandibular TFF cases (62.5 years). Therefore, with unequal variance, there is a statistically highly significant difference, which can be explained by the composition of the collective (*p* < 0.001). Regarding the first clinical sign of impending TFF, signs of TFF were documented after 3.3 days in the upper jaw and after 14.4 days in the lower jaw (Table 3). This observation is statistically significant (*p* = 0.007). Concerning further surgical procedure and removal of the necrotic graft, no statistically significant difference between maxilla and mandible was observed (Figure 11). 

## 4. Discussion

### 4.1. Rate of PFF and TFF 

Thrombosis, kinking, and spasm of the vessels have been reported as common causes of total free-flap failure in the early phase after microvascular anastomosis [32]. Venous thrombosis is more common than arterial thrombosis due to the low-flow and low-pressure venous system. Unrecognized venous thrombosis can lead to backward perfusion failure up to total stasis in the arterial system. This is followed by flap ischemia, no-reflow, and subsequent flap loss [33,34]. Fibular flaps are reported with success rates of up to 95.0% [7,14,15,16]. Study findings have shown a cumulative success rate of 88.9% (80.0% complete success and 8.9% partial flap failure). The total failure rate in the presented study is 11.1% over the last 19 years and over all types of indications for reconstruction, time of reconstruction, and method of planning (Non-VSP vs. VSP). In comparison to the here reported investigations, other reconstructive centers report total fibular flap failure rates of up to 12.4% (Table 4). Comparability of the results must be ensured concerning the chosen definition of flap success and PFF/TFF. In the presented study, strict criteria for PFF and TFF was defined. Only clear definitions will engage the collection of comparable data. Retrospective study design without standardization is often of poor data quality due to incomplete follow up data and different investigators. However, data collection over 19 years by a single investigator appears to be impractical. Loss and removal of the whole graft are clear parameters for TFF. From the clinical course and as a result of perfusion disorder through its vascular pedicle, TFF is an early flap failure. Therefore, minor, insufficient perfusion of flap elements leads to malnutrition, and thus to consecutive (sub-)total loss of skin paddle and/or bone segments. This was defined as PFF. PFF therefore does include the functional use of the flap and appears as a late flap failure. Most published data lack a differentiation between soft and hard tissue loss. Partial flap failure was reported with an incidence of 3–14% but has not been further differentiated in most cases (Table 4).

A retrospective single-center study on 129 FFF transfers over the last 20 years found TFF in 12.4% and PFF in 7.8% of cases [21]. An investigation on 20 virtual planned FFF showed that preoperative planning based on preoperative CT-scan allows to include the preoperatively planned skin paddle area [13]. Another clinical trial included preoperatively marked perforator vessels for skin paddle in digital planning and noted a survival rate of 92% (*n* = 24) in FFF transfer with three total and two partial skin paddle losses [12]. Other investigators observed seven total flap losses and three losses of skin paddle in a total of 99 FFFs [16].

In the present study, there were four losses of fibular bone segments and only two of them occurred after radiation therapy (RT). Loss of the skin paddle occurred in two cases of maxillary reconstruction and in nine cases of uni- and poly-segmental reconstruction of the mandible. After an average time of 27 days (median: 22 days, range 2–67 days), partial loss of the skin paddle was observed whereas partial bone loss occurred after mean 181 days (Median: 101.5 days, range 37–499 days). Swelling and edema are results of ongoing inflammatory processes and wound healing immediately after surgery. A critically reduced perfusion of the septo-cutaneous perforators could be the consequence. Its maximum dimension can be expected 2–3 days after surgery. The unusually late appearance of visible (sub-)total dysfunction of the skin paddle perfusion after more than the median time of 3 weeks should be interpreted with caution and as result of documentation bias. Any influence of RT in this respect can be excluded, since RT always started after PFF was already observed. The onset of adjuvant RT in the group PFF skin was at median 50.5 days (range 39–114 days) after surgery in comparison to the control group comprising complete flap success (*n* = 40), in which RT began in median 49 days (range 21–98 days) after surgery. A statistically significant difference could not be observed. However, an adjuvant RT for oncologic reasons was not to be delayed by a PFF. The results must be interpreted with caution due to the small number of cases. In the literature, the effect of prior irradiation on partial or complete loss of FFF compared with unirradiated grafts has often been reported to be statistically insignificant [38,39,40,41,42]. The effect of postoperative irradiation on partial flap failure in microvascular head and neck reconstruction has not been well described in literature and indicates that further studies are needed in this area. Verhelst et al. focused on perioperative irradiation but it was not identified as a statistically insignificant risk factor for flap failure [21]. In a study by He et al., 9 of 17 patients were irradiated postoperatively and all grafts sustained [43]. In this study, no cases of PFF were detected during or after RF. Under RT conditions, PFF could be similar to radiogenic oral mucositis, which is associated with an early inflammatory response [44,45]. These factors provide a target for biology-based mucositis-prevention strategies [46,47], and thus for PFF prevention. Further, the option of an additional skin paddle for defect closure after oncologic resection is without doubt one of the major advantages of the FFF in addition to its clinical and technical function of flap monitoring. On the other site, wound healing disorder of the donor site after skin paddle harvesting appears at a rate of 1.07–31.2% [23,48,49]. In literature, different techniques for closure of the donor site have been described [50]. Focused only on monitor function, the price for an unreliable monitor skin paddle seems to be high and should be critically reflected. Nevertheless, some authors believe that the reliability of the skin paddle for the closure of recipient site defects is insufficient in non-irradiated [51] and especially in irradiated patients [52]. In a retrospective investigation, Thome et al. observed 20% of skin paddle failures (*n* = 27) and came to a similar conclusion [53]. Other authors found a stable and sufficient vascular supply of the septo-cutaneous skin paddle by the septum intermusculare posterius and perforator vessels around the musculus soleus [54,55,56]. They emphasize the necessity of a muscle cuff around the posterior septum, which contains vessels that are crucial for skin paddle survival [24]. 

Partial bone loss may occur more frequently than previously observed and described in the literature as a result of malnutrition. Sufficient neovascularization to allow free-flap survival independent of the vascular pedicle has been reported to occur within 7 to 10 days in myo-cutaneous flaps [57,58,59]. In contrast, a comparative prospective clinical study measured hemoglobin oxygenation and capillary flow in 50 flaps (25 forearm flaps, 15 osseo-cutaneous fibular flaps, and 10 anterolateral thigh flaps) at 4 and 12 postoperative weeks. The authors found that flap autonomization rates were significantly higher in the lower jaw and non-irradiated defect sites. In addition, fascio-cutaneous flaps were found to be autonomized faster than osseo-myo-cutaneous free flaps. Myo-cutaneous flaps were never found to be autonomized after 4 weeks [60]. Kumar et al. studied blood supply of fascio-septo-cutaneous free flaps several months after surgery and found no significant blood flow through vessels across the flap inset [59]. Mücke et al. found that osseo-myo-cutaneous free flaps are significantly dependent on vascularity of the original anastomoses even 1 year after surgery [60]. According to their data, our findings should be interpreted as an adverse effect of radiation therapy (*n* = 2) and two “real” partial bone segment failures. The risk to develop osteoradionecrosis is decreased in patients with high body mass indices and on steroid therapy [61] through adequate soft-tissue bulk paired with the high-quality vascularized fibula bone [62]. Data published in the literature show that osteoradionecrosis of the original mandible occurred after a median time of 10.9 months (range 1.8–89.7 months) after RT and 90% occurred within 37.4 months [63]. In contrast, our data show bone loss after a median time of 3.38 months (range 1.23–16.63 months) and more than 6 months earlier. This observation should be interpreted with caution due to the low number of cases and should be further studied in larger study groups. 

Early TFF was incident in *n* = 3 maxillary and *n* = 17 mandible reconstructions. TFF was found after mandible reconstruction in anterior defects (Classes III-IV, 3 out of 17 cases, 17.6%) and more frequently in lateral defects (Classes I–II, *n* = 14, 82.4%). Uni- and bi-segmental were commonly used for these Classes I–II reconstructions. Reasons for increased TFF in class I and II were critical pedicle course (inner surface of mandible and mouth floor), kinking of the vascular bundle [64] and length [21,65,66]. In addition to known general risk and complicating factors, further risk factors for flap failure include postoperative swelling and edema, hematoma, movement of the neck, circular tracheal tube fixation loop, and course of the vessel through the neck [33]. 

The therapeutic procedure for partial skin or isolated bone loss depends on the jaw affected. If PFF of the skin paddle occurs in the maxilla, it is usually an uncritical situation and wound healing from peri-osseous tissue can be expected. When bone loss in the maxilla occurs, a “simple” prosthetic rehabilitation by obturator prosthetic is a therapeutic alternative [67,68] if another microvascular bone graft is not desired. Large defects can be downsized with local tissue advancement. Functional impairment (eg. scars) might be addressed in a two-staged procedure. Safe flaps routinely used in our department are the temporal myo-fascial [69], pectoralis major [70,71], and deltopectoral flaps [72]. 

In the mandible, skin paddle loss is often noncritical when the primary bone graft is vital. After wound healing local flaps and staged scars, loosing procedures can be necessary to improve dysfunction such as trismus [73]. Flaps of choice in this situation are often radial forearm [74], pectoralis major [70,71], and deltopectoral flaps [72]. When PFF of bone segments or TFF after mandible reconstructions appear, there are different strategies available that need to be evaluated in light of the patient’s condition. If patient’s condition is poor and the avital graft showed no signs of inflammation, it was left in the oral cavity (Figure 6). If inflammation and/or loosening of osteosynthesis material occurred around the avital segment, removal is necessary. Sometimes further use of osteosynthesis is possible, especially if patient-specific osteosynthesis was used. However, in the majority of cases, removal of the osteosynthesis will become necessary (Table 3). Re-osteosyntheses could be useful in combination with distant flaps like pectoralis major flaps, deltopectoral flaps, and bone hip grafts. The staged procedure of second attempts microvascular bone graft is possible after critical evaluation. The removal of the avital graft and the anticipation of stable scars building and “functional” pseudarthrosis is a further option (Figure 6).

### 4.2. Influence of Age at Flap Transfer, BMI, ASA-Score and Risk Factors in Terms of Flap Success in Relation to PFF and TFF

In this study, no significant differences between patients’ age at flap transfer and flap outcome were observed. This confirms other investigations that patients’ age at flap transfer is not crucial for flap success [75,76]. It is a surrogate parameter for the general condition of the patient [77,78]. A prospective study on 215 patients found that age ≥ 70 years had a significantly higher ASA-Score and shorter duration of surgery. Age was a risk factor for longer ICU stay and complication rate. They found no influence of age on the length of hospital stay and overall success of microvascular reconstructions [79]. In this study, more than 92% were rated ASA 2 or 3. All partial flap failures and all but one total flap occurred in both groups. ASA score and duration of the operation were found to be independent risk factors for operative revisions [76]. In literature, ASA-Rating is correlated with a higher number of postoperative complications after microvascular reconstructions [80,81] and the overall survival [82]. We calculated the relation between ‘Age at flap transfer’ and BMI and found an evenly distributed pattern. 

Concerning alcohol and tobacco abuse in the investigated group, no statistically significant differences were found. In published literature, alcohol abuse was identified as a risk factor for postoperative adverse events [82]. Tobacco abuse was shown to reduce overall survival time compared with non- and never-smokers [83,84]. A review by Van Imhoff et al. stated that survival rates are lower and recurrence rates are higher in patients who continued to smoke after having being diagnosed with Head and Neck SCC in comparison to patients who stopped smoking [85].

BMI had no statistically significant influence on flap success in this study. Low BMI or underweight at diagnosis was an independent, unfavorable prognostic factor [86,87]. Obesity was associated with better outcome and was not an independent risk factor for postoperative complications of free tissue transfer [88,89]. Other investigators found that higher BMI/obesity is a risk factor for peri- and postoperative medical complications [90].

The duration of hospitalization was calculated with a mean of 22.6 days in the PFF group and a mean of 33.8 days in the TFF group. Statistically significant differences (*p* = 0.038) were found in the occurrence of partial or total flap failures (Table 1). While the majority (68.75%, *n*= 11 out of 16) of all partial flap failures are a dysfunction of the skin paddle failures (mean duration of hospitalization partial flap loss group 20.71 days), we conclude that loss of that skin loss does not result in significantly longer hospital stay than in total flap losses. Stepwise removing of avital flap parts, deperiostizing of the bone graft, and covering with oral mucosa is an attempt for flap salvage. However, the initial stay at the ICU seems to have no statistical influence concerning upcoming PFF or TFF. It was calculated in both groups with a mean of approximately 2.0 days. 

No statistically significant difference concerning mean operating time (PFF: meantime 546 min, TFF: meantime 524 min) and flap outcome was found. Surgery time is described as a risk factor for postoperative complications [91,92,93]. Increased operating time may also be the result of a younger surgeon learning and being taught by an older instructing surgeon. However, operating time should be reduced whenever possible [92]. The study could not include ischemia time in the risk analysis because this parameter was not recorded or was incomplete.

### 4.3. PFF and TFF in Non-VSP vs. VSP and Reconstruction Methods (Immediate vs. Delayed)

The majority of isolated skin paddle losses occurred in the non-VSP group. Reasons can be seen in mechanical trauma and manipulation during free-hand transplant forming i.e., preparation of bone segments and revised application of osteosynthesis. Individually custom-made cutting guides stabilize and preserve intersegmental connectors for stabilization of several bone segments, which provide a valuable support on the vascular pedicle during transplant preparation and shaping.

No statistically significant differences were found concerning patients’ preoperative planning procedure (non-VSP vs. VSP) and time of reconstruction (immediately vs. delayed). The results confirmed the findings of other clinical investigations [94]. A retrospective study of 128 osseous free flaps with a minimum follow-up of 12 months evaluated plate-related complications in patient-specific versus conventional fixation systems. They found more complications with patient-specific plates (e.g., wound healing disorders, plate exposure, fixation failure, and subtotal osseous union) in comparison to conventional reconstruction plates, but the differences were statistically insignificant.

One possible reason for (sub-)total bone loss despite maintained perfusion of the vascular pedicle might be trauma during preparation, segmentation, and shaping of the fibula graft. Preoperatively planned and fabricated saw guides and patient-specific implants ease and accelerate the surgical procedure itself, which should be helpful in avoiding mistakes and facilitating the handling of the fibula graft. More complex shaping and osteotomization of bone segments leads to manipulation of the vascular pedicle during dissection and puts it at risk [22]. Furthermore, the impact of VSP and patient-specific plates in terms of wound healing abnormalities, plate exposure, and subtotal osseous union shows a trend towards increased complication rates compared with non-VSP with hand-bended plates. Plate-related complications were increased with radiotherapy and multi-segment flaps [95]. 

Further investigations on partial flap loss of osseomyocutaneous FFF are needed.

## 5. Conclusions

The fibula free flap constitutes a standard therapy for jaw reconstructive surgery. The present results of 180 fibula free flap over a period of 19 years shows a cumulative success rate of 88.9%, which is well comparable with other studies. The findings of our long term monocenter retrospective investigation are a position statement about flap success and partial and total loss rates, which was achieved by careful patient selection and a two team approach to reduce operating time. In partial or total flap failure, no statistically significant correlation was observed between patient age, sex, ASA, BMI, alcohol and tobacco abuse, time, and method of reconstruction (virtual versus non-virtual surgical planning). Total flap failure caused significantly prolonged hospitalization time. Partial flap failure affected mainly the skin paddle. Two-thirds of these cases were found in the non-VSP group and only two cases were observed in the virtual surgical planning group. This could be attributed to protective effects of the cutting-guide template, which possibly decrease the mechanical trauma during surgery.

## Figures and Tables

**Table 1:** Clinical details of partial (PFF; *n* = 16) and total flap failures (TFF; *n* = 20) after jaw reconstruction with fibular free flaps.

N = 36	PFF N = 16 (44.4%)	TFF N = 20 (56.6%)	*p*-Value
Age (years), SD	59.9 ± 14.4	62.5 ± 9.5	*p* = 0.520
Follow-up (months), SD	48 ± 42.9	31.5 ± 31.6	*p* = 0.193
Type of flap loss			
PFF, Skin paddle	11		
PFF, Bone segment	4		
PFF, Both	1		
Total flap loss (TFF)		20	
Sex			
Female	5	6	*p* = 0.609
Male	11	14	
Diagnosis			
Benign tumor Malignant tumor MRONJ ORN	1 15	15 2 3	
Reconstruction			
Immediate	14	17	*p* = 0.610
Delayed	2	3	
Reconstruction			
Non-VSP	10	10	*p* = 0.341
VSP	6	10	
Neck dissection			
Unilateral	11	13	n.s.
Bilateral	3	3	
None	2	4	
Tracheostomy			
None	6	8	n.s.
Primary	9	11	
Secondary	1	1	
Irradiation			
Preoperative	1	3	n.s.
Postoperative	8	4	
None	7	13	
Risk factors			
Alcohol abuse	5	9	*p* = 0.348
Tobacco abuse	9	13	*p* = 0.546
Operating time (min)	546.2 ± 94.9	524.4 ± 97.2	*p* = 0.504
Duration ICU (days)	2 ± 1.3	2.1 ± 1.7	*p* = 0.847
Hospitalization (days)	22.6 ± 9.7	33.8 ± 18.8	*p* = 0.038
BMI ≤18 18 ≤ 25 25 ≤ 30 30 ≤ 35 >35	9 3 4	1 9 7 2 1	
ASA-Score			
ASA 2	10	7	
ASA 3	6	12	
ASA 4		1	

n.s. = not significant; ORN, Osteoradionecrosis; MRONJ, Medication-related osteonecrosis of the jaw; VSP, virtual surgical planning; BMI, Body mass index; SD, standard deviation.

**Table 2:** Table depicting the locations according to the classification by Brown et al. where PFF (n=16) and TFF (n=20) occurred [29,30].

Type of Defect	PFF Skin (*n* = 11)	PFF Bone (*n* = 4)	PFF Both (*n* = 1)	TFF (*n* = 20)
Maxilla				
II	1	-	-	2
III	1	-	-	1
Mandible				
I	4	-	1	7
Ic	-	-	-	1
II	2	2	-	6
IIc	-	1	-	-
III	3	1	-	2
IV	-	-	-	1

**Table 3:** Clinical details of total flap failures (*n* = 20).

N = 20	Maxilla (*n* = 3)	Mandible (*n* = 17)	Overall (*n* = 20)	*p*
Age (years), SD	73.3 ± 1.8	60.6 ± 8.9	62.5 ± 9.5	*p* = 0.001 ^
Follow-up (months), SD	10.3 ± 9.7	35.2 ± 32.8	31 ± 31.6	
The earliest sign of flap dysfunction (days)	3. 3 ± 3.5 (Median 3)	14.4 ± 11.1 (Median 10)	12 ± 10.9 (Median 8.5)	*p* = 0.007 ^
Surgical validation and avital flap treatment procedure (days)	54 ± 60.1 (Median 36)	92.1 ± 132.0 (Median 21)	86.5 ± 123.6 (Median 22.5)	*p* = 0.449 ^
Anastomosis revisions	2	6	8	
Arterial thrombosis ^‡^	2	2	4	
Venous thrombosis ^‡^	-	4	4	n.s
Unknown	1	11	12	
Explantation of bone graft	2	16	18	
Osteosynthesis (PSI) removal	1	11	12	
Re-osteosynthesis	-	3	3	
Second flap	1	8	9	
Temporalis muscle flap	1	1	2	
Deltopectoral flap	-	1	1	
Pectoralis major flap	-	2	2	
RFF	-	1	1	
FFF	-	2	2	
Hip graft (non-DCIA)	-	1	1	

**^‡^** Kind of thrombosis was evaluated during microsurgical revision. (n.s. = not significant; ^ Equal variances not assumed; DCIA, deep circumflexia iliac artery; PSI, patient-specific implant; SD, standard deviation; RFF, radial forearm flap).

**Table 4:** Overview of FFF total and partial flap failure rates in the literature.

Authors	Investigation Period	*n*	Total Flap Failure	Partial Flap Failure
This study	2002–2020	180	11.1%	8.9%
Colletti et al. [16]	2002–2010	99	7%	3%
Gallegos-Hernandez et al. [35]	1996–2006	87	16.1%	-
Götze et al. [12]	2013–2015	24	12.5%	8.3%
Lopez-Arcas et al. [36]	1992–2006	117	-	8.5%
Momoh et al. [23]	2005–2009	157	1%	14%
Mücke et al. [37]	2009–2013	76	9.2%	-
Seruya et al. [22]	2003–2012	68	4.41%	11.76%
Shroff et al. [14]	2009–2013	30	6.66%	-
Verhelst et al. [21]	1996–2016	129	12.4%	7.8%

## Data Availability

The data presented in this study are available upon request from the corresponding author.
